# Associations between childhood threat and deprivation experiences, self- and other-mentalizing, and adult psychopathology: evidence from a community sample

**DOI:** 10.3389/fpsyt.2026.1745165

**Published:** 2026-07-17

**Authors:** Flora Descartes, Karyn Doba, Emilie Auger, Vincent Besch, Nader Perroud, Martin Debbané, the Réseau Francophone des Thérapies Basées sur la Mentalisation (RF-TBM) Consortium

**Affiliations:** 1 Faculty of Psychology and Educational Sciences, University of Geneva, Geneva, Switzerland; 2 CNRS, UMR 9193-SCALab, Cognitive and Affective Sciences, University of Lille, Lille, France; 3 Division of Institutional Measures, Department of Psychiatry, Geneva University Hospitals, Geneva, Switzerland; 4 Faculty of Medicine, University of Geneva, Geneva, Switzerland; 5 Research Department of Clinical, Educational and Health Psychology, University College London, London, United Kingdom

**Keywords:** adult psychopathology, childhood trauma, deprivation, threat, mentalization, self- and other-mentalizing

## Abstract

**Introduction:**

Childhood adversity is a well-established transdiagnostic risk factor for psychopathology, yet the mechanisms linking specific adversity types to mental health remain unclear. The Dimensional Model of Adversity and Psychopathology (DMAP) distinguishes deprivation, defined as the absence of expected social and cognitive inputs, from threat, defined as exposure to violence or danger, and links them to impairments in cognitive and socio-emotional development. These forms of adversity may critically impact socio-cognitive mechanisms such as mentalization, the capacity to interpret one’s own and others’ behavior in terms of mental states. Mentalization may thus represent a key pathway through which adversity dimensions confer risk or protection for psychopathological expression.

**Methods:**

A community sample of 302 adults (53% female; M = 37.1, SD = 12.3) completed validated self-report measures of childhood trauma (CTQ), self- and other-mentalizing (MentS), and psychological symptoms (Global Severity Index, SCL-90-R). Partial Least Squares Structural Equation Modeling (PLS-SEM) examined direct and indirect pathways linking deprivation and threat to psychological symptoms, associated with self- and other-mentalizing. Supplementary analyses tested age and gender moderation.

**Results:**

The measurement model showed good reliability and validity. The structural model explained 36.8% of the variance in psychopathological expression. Deprivation was negatively associated with other-mentalizing. Threat was positively associated with other-mentalizing and negatively with self-mentalizing. Self-mentalizing was strongly and negatively related to psychopathology, whereas other-mentalizing showed positive associations with symptomatology and self-mentalizing. A significant indirect path emerged from threat to other-mentalizing through self-mentalizing to psychopathology. Age moderated the self-mentalizingpsychopathology link.

**Conclusion/discussion:**

Findings support DMAP and mentalization-based frameworks, indicating that threat and deprivation exert distinct effects on mentalization and psychopathology. Self-mentalizing functions as a protective factor, whereas difficulties in understanding others reflect compensatory but maladaptive adaptations to adversity. Together, these results identify mentalization as a transdiagnostic mechanism linking early adversity with adult psychological functioning.

## 1 Introduction

Childhood adversity remains a pervasive global issue, affecting emotional, cognitive, and social development across the lifespan. According to UNICEF, nearly 400 million children worldwide regularly experience violent discipline at home (UNICEF, press release, 11 June 2024). Beyond physical violence, many individuals grow up in environments characterized by deprivation, with one in five aged 2 to 4 years not engaging in play with caregivers, and one in seven under age 5 lacking toys or play materials (United Nations Children’s Fund, 2025). These indicators reflect that early adversity can encompass a wide spectrum of experiences, including threatening and sometimes violent interactions in the physical, psychological, emotional, and sexual domains, but also deprivation from the early experiences necessary to satisfy basic needs (1). Collectively, such experiences have been conceptualized under broad terms such as Adverse Childhood Experiences (ACEs) (2) or Early Life Stress (3, 4).

A large body of research has shown that exposure to childhood adversity, particularly when chronic or multifaceted, is associated with lasting impairments in multiple domains of functioning, including neurobiological, emotional, cognitive, and relational processes (5–7). Historically, such enduring effects have been described as type II trauma (8, 9), reflecting the cumulative and relational nature of prolonged exposure to threat and deprivation. In line with this perspective, the ICD-11 has introduced the diagnosis of complex post-traumatic stress disorder (C-PTSD) to capture the long-term psychological and interpersonal consequences of repeated or chronic trauma (10, 11). Beyond diagnostic frameworks, evidence suggests that trauma exposure and its psychological effects occur along a continuum shaped by factors such as chronicity and relational dependency such as victimization by a primary caregiver, highlighting that trauma-related symptoms can affect anyone, albeit to varying degrees (12–15). Early adversity has been linked to a broad spectrum of psychopathological outcomes, including post-traumatic stress disorder (PTSD), depression, anxiety, and both internalizing and externalizing symptoms (16–18). Together, these findings support the view that trauma constitutes a transdiagnostic risk factor, contributing to diverse forms of psychopathology rather than mapping onto a single diagnostic category (19–21). This reinforces the need to investigate trauma-related processes in the general population, beyond purely clinical contexts.

To advance the understanding of how early adversity gives rise to this diversity of outcomes, McLaughlin and colleagues proposed the Dimensional Model of Adversity and Psychopathology (DMAP), or threat and deprivation framework (3, 21). The DMAP distinguishes between two core, partially overlapping dimensions of adversity: deprivation, which refers to the absence of expected social and cognitive inputs, and threat, which refers to exposure to events that threaten bodily integrity or safety. This framework moves beyond cumulative risk approaches, which treat adversity as a single, additive factor, by emphasizing qualitative differences in how distinct forms of early stress affect development. Drawing primarily on evidence from animal studies on sensory deprivation and fear learning and corresponding human studies, findings suggest that these dimensions engage different neurodevelopmental pathways (3). Specifically, deprivation, encompassing experiences such as neglect, limited stimulation, and reduced caregiver interaction has been associated with altered cortical development, particularly in associative regions that support complex cognitive and social processes (4). Based on their own research, Sheridan and McLaughlin (4) report that children reared in deprived environments, such as institutions or neglectful homes, exhibit thinner association cortices and reduced prefrontal activation, which correspond to lower performance in tasks requiring abstract reasoning, language, and social understanding. In contrast, threat-inducing experiences, including exposure to abuse, violence, or chronic fear, have been linked to alterations in neural systems underlying emotional learning and regulation, such as the amygdala, hippocampus, and ventromedial prefrontal cortex. These changes manifest in heightened emotional reactivity, attentional biases toward threat, and difficulties regulating affective states. The authors suggest that while the two dimensions often co-occur, their distinct neurocognitive signatures may differentially shape vulnerability to later psychopathology.

Within the DMAP perspective but not explicitly mentioned, mentalization emerges as a potential psychological mechanism which may inform how deprivation and threat-inducing experiences link to later socio-emotional and psychopathological outcomes. Mentalization refers to the capacity to interpret one’s own and others’ behavior in terms of underlying mental states such as beliefs, desires, intentions, and emotions (22). It is operationalized as a multifaceted construct integrating cognitive (e.g. perspective taking or belief-desire reasoning) and affective (grounds for mentalization in an affectively felt reality) dimensions, self- and other-oriented processes, and automatic and controlled forms of processing (23, 24). Importantly, these components may map closely onto the domains shown to be sensitive to deprivation and threat. For instance, cognitive mentalization may depend on enriched social-cognitive input, whereas affective mentalization may be particularly vulnerable to experiences of threat and emotional dysregulation. Empirical evidence substantiates these associations. Meta-analytic findings indicate that childhood maltreatment is associated with lower mentalizing (25) and clinical research suggests that impairments in both self- and other-mentalizing are central to trauma-related psychopathology, including borderline personality disorder and dissociative conditions (26, 27). Finally, the mentalization framework is closely linked to large scale preventive interventions in children (28) but also specialized therapeutic interventions (26, 29) for individuals dealing with adverse experiences.

Although mentalization is a multidimensional construct encompassing different components, the present study focuses specifically on the distinction between self- and other-oriented mentalizing. This distinction reflects a relational and developmental perspective; whereby early adverse experiences may differentially shape how individuals understand their own mental states and those of others. Importantly, recent measurement advances, such as the Mentalization Scale (MentS) (30), now allow self- and other-oriented mentalizing to be examined separately. This methodological development opens new possibilities for exploratory research in the field of developmental trauma, where such distinctions have been theoretically emphasized but less frequently operationalized. This choice of dimension reflects how the mentalization framework puts forward the hypothesis that adverse caregiving environments characterized by deprivation and/or threat can significantly shape the ways individuals think about and learn from others’ minds (31). Indeed, from this point of view, adverse experiences, especially from caregivers, alters the experience of (not) being mentalized, and alter interpretations of one’s own mental states. In turn, being exposed to a potentially threatening caregiver can translate into hypervigilance to and/or avoidance of others’ mental states (24, 32). In line with these hypotheses, a recent study found that the tendency of mistrusting information coming from others (epistemic mistrust) fully mediated the relationship between childhood maltreatment and ineffective self-mentalizing (33). Another study reported that child sexual abuse was found to be a significant contributor to children’s mentalizing of others (34). Accordingly, the present study adopts an exploratory approach to examine whether threat and deprivation are differentially associated with self- and other-oriented mentalizing, without assuming specific directional or causal relationships. Overall, meta-analytical evidence indicates that childhood maltreatment is significantly associated with alterations in the development of mentalizing capacities (25). Additionally, the present study examines a specific pathway going from early adverse childhood experiences to other-mentalizing, to self-mentalizing, to psychopathological expression. This specification is informed by developmental models emphasizing the interpersonal origins of mentalization within attachment relationships, where caregivers, and more broadly family members, peers, and the social environment, provide marked mirroring of the child’s internal states supports the development of self-reflective capacities (24, 35).

At the neurobiological level, the neural regions sustaining self- and other-mentalizing partially overlap in a broad and non-specific manner with cortical and subcortical regions specifically impacted by developmental deprivation and threat (24, 3, 4). Deprivation-related experiences affect global morphological indices such as cortical thickness, but also relate to posterior and lateral brain areas such as the temporo-parietal junction which is key in self-other differentiation of mental states to navigate complex social interactions (36, 37). Threat-related impacts on cerebral development potentially alter limbic-prefrontal circuits sustaining affective and self-referential components (3, 24, 38). Importantly, these neural findings do not imply a one-to-one correspondence with specific mentalization dimensions but rather indicate that early adversity may influence broader affective and socio-cognitive systems that are theoretically relevant to mentalization processes. This convergence therefore suggests that early adversity may impair integrative socio-cognitive capacities such as mentalization by disrupting the balance and connectivity of these neural systems (24). For instance, disruptions in trusting others and impairments in mentalizing were found to be risk factors for psychological maladjustment in a community sample of adolescents (39). A more specific understanding of the differential roles of self- and other-mentalizing patterns in relation to early adverse experiences may shed light on how developmental mechanisms sustaining later psychopathological symptoms.

Building on the integration of the DMAP and mentalization frameworks, the present study examines the associations between deprivation and threat and the two dimensions of mentalization, self- and other-oriented mentalizing, and investigates how these processes relate to psychological symptoms in a non-clinical sample. This study adopts an exploratory approach to examine (a) whether threat and deprivation are differentially associated with self- and other-mentalizing, and (b) how variations in these mentalization dimensions relate to overall psychological distress.

## 2 Methods

### 2.1 Participants and procedure

This study comprised a community sample of N = 302 adult community participants, comprising 161 females aged between 19 and 75 years (M = 37.1, SD = 12.3). Participants were recruited on the Prolific website during the month of March 2022 and they provided informed written consent. Their data was fully anonymized. Inclusion criteria were to be older than 18, to be fluent in French, and to have never been hospitalized for psychiatric reasons. To ensure the quality of the data they provided, several inclusion and exclusion controls were applied. Participants were required to have satisfactorily submitted at least 15 online surveys beforehand to ensure a good level of use of the online platform and reduce data entry errors. Nonsensical items were randomly inserted in the survey and response times were monitored to check for participants’ understanding and attention (40). Participants were excluded if they provided incorrect responses to more than two bogus items or if their total response time was faster than the sample mean by more than two standard deviations (i.e., > 2 SD below the mean). In addition, participants who gave only one incorrect response on bogus items were also excluded if their total response time was at least one standard deviation below the sample mean. As a result of the combination of these rules, N = 70 participants were excluded, which resulted in a final sample of 302 participants. The study was approved by the Swiss Ethics Commission in Geneva under project id 2021-01100.

### 2.2 Measures

2.2.1 *The Childhood Trauma Questionnaire - Short Form (CTQ-SF; *(41)) is a 28 item, 5-point Likert scale from 1 to 5, brief screening instrument designed to assess histories of maltreatment. Three items are non-clinical, used to evaluate denial and minimization. Through five subscales scores (theoretical score range from 5 to 25 for each subscale), it evaluates the presence and severity of physical, emotional, and sexual abuse, as well as emotional and physical neglect. The French version of the CTQ-SF has been validated with good psychometric properties suitable for both clinical and research purposes (42).

Childhood trauma severity levels for each type of abuse and neglect were categorized based on established recommended cut-off scores which classify responses into four categories: none, low, moderate, and severe. Higher scores indicate more severe exposure to childhood trauma type (41). In line with prior literature, participants were considered to have experienced maltreatment if their scores met or exceeded the threshold for “moderate to severe” levels (43). The cut-off values are as follows: emotional abuse (≥ 13), physical abuse (≥ 10), sexual abuse (≥ 8), emotional neglect (≥ 15), and physical neglect (≥ 10).

To align with dimensional models of early adversity, the subscales of the Childhood Trauma Questionnaire (CTQ) were grouped into two higher-order categories reflecting the constructs of threat and deprivation. The threat dimension included subscales related to abuse, specifically emotional abuse, physical abuse, and sexual abuse. In contrast, the deprivation dimension comprised emotional and physical neglect. This categorization is consistent with theoretical frameworks distinguishing between experiences that signal danger to the individual (threat) and those that reflect the absence of expected cognitive and social inputs (deprivation) (3).

In the present sample, the Childhood Trauma Questionnaire (CTQ) subscales showed acceptable to excellent reliability: Emotional Abuse (α = 0.880), Physical Abuse (α = 0.853), Sexual Abuse (α = 0.940), Emotional Neglect (α = 0.906), and Physical Neglect (α = 0.714).

Pearson correlations among the Childhood trauma Questionnaire subscales in the present study were computed to assess the coherence and discriminant validity of the constructs. All subscales were moderately correlated (ranging from r = 0.26 to 0.67).

2.2.2 *The Mentalization Scale (MentS*; (30)) is a self-report questionnaire, 5-point Likert scale from 1 to 5, designed to assess the capacity to mentalize. The scale measures various dimensions of mentalization through three subscales, namely the Motivation to mentalize, mentalizing Others and mentalizing the Self. Only the self- (theoretical score range 8-40) and other-mentalizing (theoretical score range 9-45) subscales were included in the present study. The francophone validation of the MentS excluded one item (item 25) from the scoring procedure and demonstrated acceptable-to-good internal consistency for the control sample for the Other (α = 0.792) and Self (α = 0.824) subscales) (44).

The sample used in the present study is the same as the one used in the French validation of the MentS scale. Hence, the alpha coefficients are the same. Pearson correlations between subscales were of r = 0.175 between the self- and other-mentalizing subscales supporting their theoretical relatedness without indicating redundancy.

2.2.3 *The Symptom Checklist-90-Revised (SCL-90-R; (*45*)):* is a 90-item, self-report questionnaire that utilizes a 5-point Likert scale with a theoretical score range from 0 to 4. It assesses nine primary symptom dimensions: Somatization, Obsessive-Compulsive, Interpersonal sensitivity, Depression, Anxiety, Hostility, Phobic Anxiety, Paranoid Ideation and Psychoticism. Additionally, it provides three scores reflecting global distress: Global Severity Index (GSI), Positive Symptom Distress Index and Positive Symptom Total. This scale has been validated among French-speaking populations (46). Only the Global Severity Index was included in the present study.

In the present sample, the Global Severity Index (GSI) showed excellent reliability (α =0.984). These results support the internal consistency of all measures used in the study.

### 2.3 Statistical analysis

Descriptive statistics (means, standard deviations, and frequency distributions), linearity checks, and reliability analysis were computed using IBM SPSS Statistics (version 29.0.2.0, build 20). This software was used to summarize sample characteristics and questionnaire scores prior to structural modeling.

In line with the standard specification of Partial Least Squares Structural Equation Modeling (PLS-SEM), the structural relationships between latent variables were modeled as linear (47). Linearity assumptions were tested using ANOVA-based procedures, examining both the linear component and the deviation from linearity for each hypothesized relationship. A significant p-value (<.05) for the linear component indicates the presence of a statistically significant linear relationship, whereas a non-significant deviation from linearity (p ≥.05) suggests no statistically detectable departure from linearity. Scatterplots of the PLS latent variable scores for each structural path were visually inspected to identify potential non-linear patterns.

A PLS-SEM approach was employed using SmartPLS software (version 4.1.1.4) to estimate both the measurement (outer) and structural (inner) models (48). PLS-SEM is a variance-based SEM technique particularly suited to prediction-oriented research involving complex indirect associations, moderate sample sizes, and data that may deviate from multivariate normality (47, 49). Reflective measurement models were specified for threat and deprivation latent constructs, consistent with their conceptualization as indicators reflecting broader underlying dimensions, treating abuse and neglect subtypes as manifestations of broader threat and deprivation dimensions (47). For the mentalizing dimensions and psychopathology, the construct was specified as a reflective single-indicator construct, given its unidimensional conceptualization.

Finally, a rule of thumb for PLS-SEM estimations is that the sample size should be equal to the larger of the following (50): 10 times the number of indicators of the scale with the largest number of manifest indicators. Accordingly, given the sample size in the current study, the PLS-SEM analyses were feasible for the present sample.

In what we will refer to as Model 1, the direct and indirect relationships between two distinct childhood adversity dimensions and psychological symptoms outcomes were examined. Specifically, the model examined how two dimensions of childhood adversity influence adult psychological outcomes. The first, threat, was a latent construct indicated by the following manifest variables: physical, sexual, and emotional abuse. The second, deprivation, was assessed through two manifest variables: physical and emotional neglect. Both dimensions were tested for their impact on the latent construct of adult psychopathological expression, with overall psychological distress as manifest variable. The model also incorporated two mediating latent variables, self-mentalizing and other-mentalizing, each with their respective manifest variables of the same name. Finally, the model assessed a specific targeted mediating pathway from early adversity to other-mentalizing to self-mentalizing to psychopathological symptoms. This directional specification was theoretically motivated by developmental models of mentalization, which emphasize the fundamentally interpersonal origins of reflective functioning. This structure enabled the examination of both direct pathways from early adversity to psychopathological symptoms and indirect pathways mediated by specific aspects of mentalization.

To evaluate the statistical significance of the structural model, a non-parametric bootstrapping procedure with 10,000 resamples was performed. This approach allows for the estimation of standard errors and confidence intervals without assuming normality, which is consistent with the assumptions of PLS-SEM (Hair et al., 2017). Bootstrapping was used to test the significance of direct and indirect effects between constructs. Bias-corrected confidence intervals were applied to determine whether the path coefficients differed significantly from zero.

### 2.4 Supplementary analyses

To examine the potential moderating effects of key demographic variables, supplementary analyses were conducted. In Model 1, gender differences were tested using a permutation-based multigroup analysis (MGA), which allows for the comparison of structural path coefficients between male and female subgroups (51). Additionally, the moderating effect of age was tested through interaction terms computed between age and the relevant latent predictor constructs: self-mentalizing referred to as Model 2, and other-mentalizing referred to as Model 3.

Finally, to assess the directional robustness of the structural model, an additional reverse path analysis was conducted in what will be referred to as Model 4. Specifically, an alternative model was tested in which the directional link between the self-mentalizing and other-mentalizing constructs was inverted, relative to the originally hypothesized model. This procedure was implemented to evaluate whether the theoretical direction of influence, from other- to self-mentalizing was better supported than the reverse (from self- to other-mentalizing). Such a test helps to rule out misspecification due to potentially reciprocal or ambiguous causal pathways and supports the theoretical justification for the proposed directionality. All tested models and the corresponding path coefficients are presented in the **Supplementary Material**.

## 3 Results

### 3.1 Descriptive statistics

The community sample consisted of 302 participants, including 161 females and 141 males. The average age of female participants was 37.3 years (SD = 13.6), with ages ranging from 19.6 to 74.9 years. Male participants had a mean age of 36.8 years (SD = 10.7), with a minimum age of 20.2 and a maximum of 73.4 years. The median age was 34.64, with 50% of participants between 25.98 and 46.42 years old (interquartile range = 20.44). There were no missing data in the study sample.

Emotional neglect had the highest mean score (M = 12.50, SD = 5.13), followed by emotional abuse (M = 9.57, SD = 4.89). Detailed scores are found in **Table 1**. As shown in **Table 2**, based on the recommended cut-offs, 24.17% of participants reported emotional neglect, 16.56% reported emotional abuse, and smaller proportions met criteria for physical abuse (9.60%), physical neglect (11.59%), and sexual abuse (5.63%).

**Table 1:** Manifest and latent variables included in model 1.

Latent variables	Manifest variables	Measure	M (SD)	Range	Weight
Deprivation	Emotional neglect	CTQ	12.50 (5.13)	5-25	.642
	Physical neglect		7.55 (3.18)	5-22	.485
Threat	Physical abuse	CTQ	6.87 (3.34)	5-25	.297
	Emotional abuse		9.57 (4.89)	5-25	.571
	Sexual abuse		5.96 (2.96)	5-25	.352
Self-Mentalizing	Self-mentalizing	MentS-Self	26.6 (6.61)	8-40	1
Other-Mentalizing	Other-mentalizing	MentS-Others	33 (5.27)	18-45	1
Psychopathology	Overall psychological distress	SCL90-GSI	0.71 (0.68)	0.00-3.08	1

M, Mean; SD, Standard Deviation; CTQ, Childhood Trauma Questionnaire; MentS, Mentalization Scale; SCL90-GSI, Symptom Checklist 90-items Global Severity Index. Significant weights are presented for Model 1 with 8 manifest variables on 5 latent variables.

**Table 2:** Childhood trauma exposure in the total sample (N = 302).

Category	n	%
Trauma type		
Emotional abuse	50	16.56
Physical abuse	29	9.60
Sexual abuse	17	5.63
Emotional neglect	73	24.17
Physical neglect	35	11.59
Number of trauma types		
0	194	64.24
1	57	18.87
2	22	7.28
3	16	5.30
4	10	3.31
5	3	0.99

Percentages are based on the total sample (N = 302). Trauma categories are not mutually exclusive. CTQ cutoff scores: emotional abuse ≥ 15, physical abuse ≥ 12, sexual abuse ≥ 12, emotional neglect ≥ 17, and physical neglect ≥ 12.

Among the 302 participants, 86 reported exposure to deprivation-related trauma and 64 to threat-related trauma. Co-occurrence was substantial, with 42 participants reporting exposure to both dimensions. Overall, 108 participants reported at least one type of childhood adversity, whereas 194 reported none.

Mentalization subscale scores were as follows: MentS-Others (other-mentalizing) (M = 33.0, SD = 5.27, range = 18 - 45), and MentS-Self (self-mentalizing) (M = 26.6, SD = 6.61, range = 8 - 40).

The Global Severity Index (GSI), derived from the SCL-90, was used to assess overall psychological distress. The GSI scores ranged from 0.00 to 3.08 (M = 0.71, SD = 0.68). This indicates a moderate level of symptom reporting (45), with considerable variability among participants.

Descriptive statistics are shown in **Table 1**.

### 3.2 PLS-SEM modeling

Tests of linearity assumptions yielded the following results: the linear component was statistically significant for Deprivation with Psychopathology (F = 16.97, p <.001), Deprivation with Self-Mentalizing (F = 5.16, p = .024), Threat with Psychopathology (F = 29.24, p <.001), Threat with Self-Mentalizing (F = 5.28, p = .022), Self-Mentalizing with Psychopathology (F = 129.35, p <.001), and Other-Mentalizing with Self-Mentalizing (F = 8.18, p = .005). Deviation from linearity was significant for the relation between Threat and Psychopathology (F = 1.53, p = .005) and the interaction between Other-Mentalizing and Self-Mentalizing (F = 1.54, p = .041). While deviations from linearity were statistically significant for two relationships, in conjunction with the significant linear components and in the absence of clear curvilinear patterns in the scatterplots of latent variable scores, these results support the adequacy of a linear approximation for the structural relationships examined.

The following results in the outer and inner models are based on a non-clinical community sample and should therefore be interpreted as reflecting patterns of association within the general population, rather than clinical-level psychopathology.

#### 3.2.1 Outer model - Model 1

The standardized outer weights for the manifest variables of each latent construct are shown in **Table 1**. The reflective measurement model for PLS-SEM analysis was evaluated using standard criteria for indicator reliability, internal consistency, convergent validity, and discriminant validity. All outer loadings exceeded the recommended threshold of 0.70, indicating satisfactory indicator reliability, except for sexual abuse with a value of 0.695, which was marginally below the cutoff but retained due to theoretical relevance (52). Composite reliability values (ρc) ranged from 0.849 (threat) to 1.000 (other- and self-mentalizing and psychopathology), and Cronbach’s alpha values ranged from 0.724 (deprivation) to 1.000, demonstrating good internal consistency.

Following established guidelines for PLS-SEM measurement models (52–54), convergent validity was supported by Average Variance Extracted (AVE) values, all of which exceeded the recommended 0.50 threshold (e.g., AVE = 0.781 for deprivation; AVE = 0.654 for threat). Discriminant validity was confirmed through Heterotrait-Monotrait (HTMT) ratios, all of which remained below the threshold of 0.90 (e.g., HTMT = 0.866 for threat with deprivation; HTMT = 0.072 for other-mentalizing with deprivation). Outer variance inflation factor (VIF) values ranged between 1 and 1.888, hence below conservative threshold of 3, indicating no concerns with multicollinearity. Overall, these results provide robust evidence for the reliability and validity of the reflective measurement model.

#### 3.2.2 Inner model - model 1

**Figure 1:**
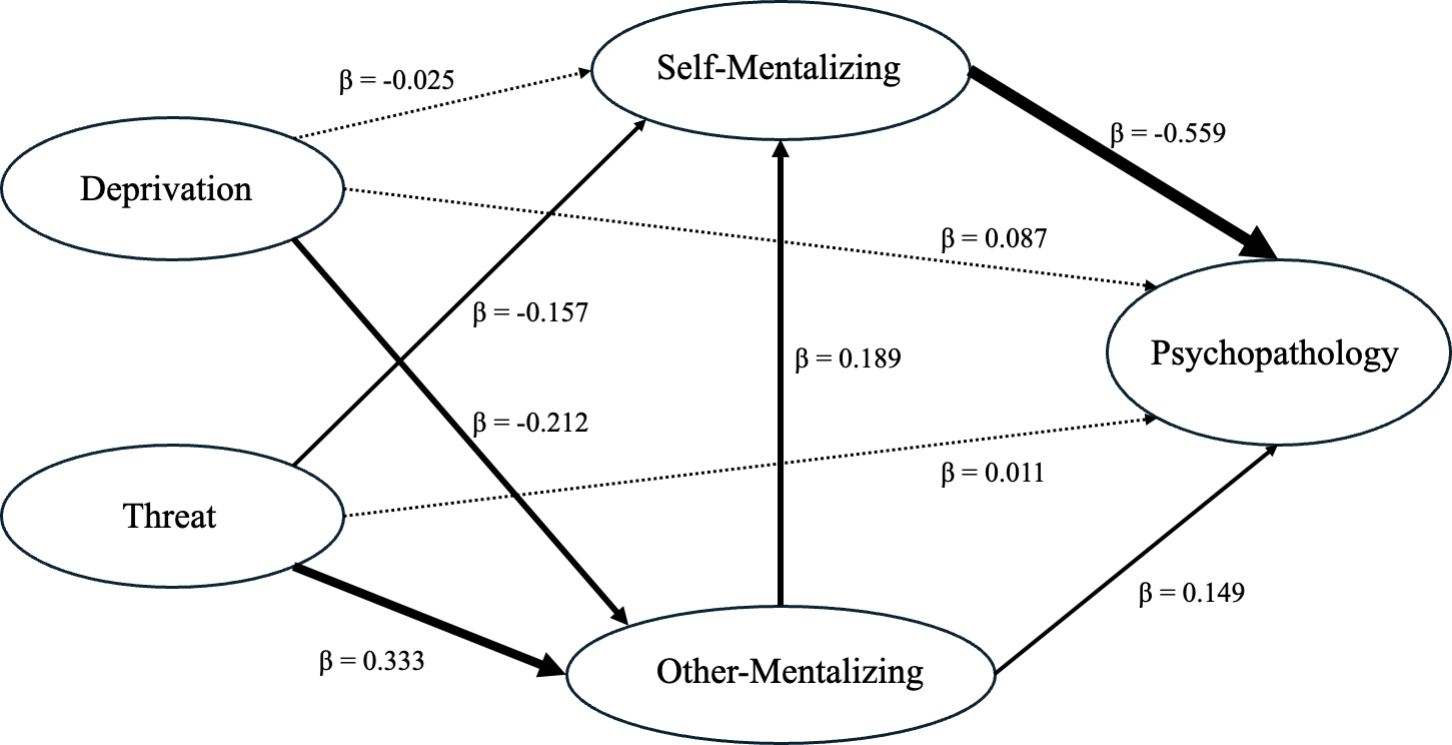
Model 1 - with standardized path coefficients. Dashed lines indicate non-significant relationships. β represents standardized path coefficients; The thickness of the arrows is proportional to the strength of the standardized path coefficients (β).

**Figure 1** shows the final multiple mediation model with standardized path coefficients of direct effects.

All inner VIF values ranged between 1.067 and 1.906, remaining well below the conservative threshold of 3, thereby indicating the absence of multicollinearity issues (55). Regarding explained variance, the model accounted for 36.8% of the variance in psychopathology, 5.7% in other-mentalizing and 4.5% in self-mentalizing. Lastly, the model demonstrated an acceptable level of global fit, with a Standardized Root Mean Square Residual (SRMR) value of 0.076, remaining below the recommended threshold of 0.08 (54).

Several direct paths emerged as statistically significant and direct bootstrapped path coefficients are reported in **Table 3**. Concerning direct effects from deprivation, it was significantly and negatively associated with other-mentalizing (β = -0.212, 95% CI [-0.362, -0.049]). Deprivation was not significantly associated with self-mentalizing (β = -0.025, 95% CI [-0.190, 0.131]), nor with psychopathology (β = 0.087, 95% CI [-0.032, 0.212]). Concerning direct effects from threat, it was significantly and positively associated with other-mentalizing (β = 0.333, 95% CI [0.184, 0.471]) and negatively with self-mentalizing (β = -0.157, 95% CI [-0.299, -0.014]). The direct effect from threat to psychopathology was not significant (β = 0.111, 95% CI [-0.005, 0.230]).

**Table 3:** Direct bootstrapped path coefficients for model 1.

Direct effects between latent variables	β mean (SD)	95% bootstrap CI
Deprivation → other-mentalizing	-0.212 (0.080)	[-0.362, -0.049] *
Deprivation → self-mentalizing	-0.025 (0.081)	[-0.190, 0.131]
Deprivation → psychopathology	0.087 (0.063)	[-0.032, 0.212]
Threat → other-mentalizing	0.333 (0.073)	[0.184, 0.471] *
Threat → self-mentalizing	-0.157 (0.073)	[-0.299, -0.014] *
Threat → psychopathology	0.111 (0.060)	[-0.005, 0.230]
Other-mentalizing → self-mentalizing	0.189 (0.067)	[0.055, 0.317] *
Other-mentalizing → psychopathology	0.149 (0.050)	[0.052, 0.245] *
Self-mentalizing → psychopathology	-0.559 (0.039)	[-0.631, -0.477] *

CI, confidence interval; *p < 0.05.

Concerning direct effects from other-mentalizing, it was significantly and positively associated with self-mentalizing (β = 0.189, 95% CI [0.055, 0.317]). Other-mentalizing was significantly and positively associated with psychopathology (β = 0.149, 95% CI [0.052, 0.245]). Finally, self-mentalizing had a strong negative direct effect on psychopathology (β = -0.559, 95% CI [-0.631, -0.477]).

Concerning indirect effects in Model 1, three paths from threat to psychopathology were significant. First, threat had a significant indirect effect on psychopathology through self-mentalizing (β = 0.088, 95% CI [0.008, 0.169]), and second, through other-mentalizing (β = 0.049, 95% CI [0.015, 0.092]. Third, threat showed a significant indirect effect sequentially via other-mentalizing and self-mentalizing leading to psychopathology (β = -0.035, 95% CI [-0.069, -0.009]). Indirect effects from deprivation to psychopathology were all non-significant: via self-mentalizing (β = 0.014, 95% CI [-0.073, 0.107]), via other-mentalizing (β = -0.032, 95% CI [-0.068, -0.005]), and via the sequential pathway from deprivation to other-mentalizing to self-mentalizing to psychopathology (β = 0.022, 95% CI [0.003, 0.049]).

### 3.3 Supplementary analyses

To assess potential gender differences in Model 1, a permutation multi-group analysis (MGA) was conducted. Results revealed no significant gender differences in direct effects. Differences ranged from Δβ = -0.161, 95% CI [-0.275, 0.278], p = .252 for the association between other- and self-mentalizing to Δβ = 0.090, 95% CI [-0.311, 0.325], p = 0.593 for the association between deprivation and self-mentalizing. Similarly, no significant group differences were observed for the association between deprivation and psychopathology (Δβ = 0.089, 95% CI [-0.266, 0.250], p = .495), and between mentalization of the self and psychopathology (Δβ = -0.071, 95% CI [-0.150, 0.149], p = .378), nor for any of the remaining direct associations.

Model 2 was estimated to examine the moderating effect of age on the relationship between self-mentalizing and psychopathology. Bootstrapped analyses indicated a significant moderation effect (β = 0.097, 95% CI [0.013, 0.195], p = .031), such that the negative association between self-mentalizing and psychopathology was weaker among older participants. Model 3 was then estimated to test the moderating effect of age on the relationship between other-mentalizing and psychopathology. No significant moderation effect was observed (β = 0.005, 95% CI [-0.085, 0.090], p = .912). To assess the directional robustness of the proposed model, a reverse path analysis was conducted, testing the inverse directional link from threat to self- to other-mentalizing to psychopathology in what is referred to as Model 4. In this model, the indirect path from threat to psychopathology via self- then other-mentalizing was not significant (β = -0.003, 95% CI [-0.010, 0.000]). Compared with the original directional Model 1, these results indicate that the double mediation pathway from threat to psychopathology, successively mediated by self- and then other- mentalizing has not reached statistical significance.

## 4 Discussion

The present study examined how two forms of childhood adversity, namely deprivation and threat, relate to adult psychopathology through self- and other-mentalizing processes. The results from our structural equation modeling analyses shed light on both direct and indirect paths linking early adverse experiences, mentalizing and psychopathological expression. The resulting model explained a substantial proportion of variance and demonstrated good overall fit. In the following, we will begin by discussing the direct path effects, followed by the indirect path effects. We will then address gender and age effects, clinical implications, and, finally, the study’s limitations and directions for future research. The present findings should be interpreted in the context of a non-clinical community sample and therefore reflect associative patterns relevant to the general population, rather than mechanisms specific to clinical disorders. Moreover, the cross-sectional nature of the data limits conclusions about the directionality of the observed associations.

Examining the direct effects, we found that early deprivation was associated with a reduced capacity to mentalize others. This result is in line with the notion of the pedagogical stance offered by caregivers, that is, the degree to which parents engage the child in shared attention and joint meaning-making activities (56, 57). When such scaffolding is limited, children may receive fewer opportunities to learn about others’ intentions and emotions, which in turn limits their curiosity and exploration of the social world. This interpretation aligns with longitudinal neurodevelopmental findings showing that deprivation is associated with alterations in higher-order associative regions, including the temporoparietal junction and medial prefrontal cortex, key areas for theory of mind and social-cognitive processing, across childhood and adolescence (37, 58). In this context, the absence of a significant link between deprivation and self-mentalizing may require further inquiry. We hypothesize that the distribution of deprivation may have been too constrained in this community sample, where multiple and severe neglect experiences may be less represented. Such associations may become more significant in high-risk or clinical samples where deprivation is more pervasive and compounded with broader environmental constraints such as socioeconomic adversity (59).

Concerning early threat experiences, our model revealed both a positive and significant relationship with other-mentalizing, as well as a negative significant negative association with self-mentalizing. This dual pattern of heightened other-mentalizing and reduced self-mentalizing is in line with the mentalization framework, particularly with the notion that high-intensity experiences disrupt the delicate balance underlying the integration of mentalizing processes (60). Furthermore, these results align with the latent vulnerability framework (61), which argues that early threat exposure may calibrate neurocognitive systems toward heightened vigilance to others’ intentions as an adaptation enhancing short-term safety, to the expense of undermining self-directed internal emotional awareness. According to this model, this hyper-attunement to external cues can become maladaptive, fostering social mistrust and emotional dysregulation, and thus increasing vulnerability to psychopathology.

We further note the direct effect linking other- to self-mentalizing. Our results are consistent with a developmental account of mentalizing in which representations of others may scaffold self-awareness, although the cross-sectional nature of the data precludes conclusions about directionality and leaves open the possibility of reciprocal influences across development (35, 62). This view is in line with work by Fotopoulou and Tsakiris (63) on the development of the self and its bodily and social origins: *“Understanding others requires a ‘good enough’ representation of one’s own (interoceptive) states, because our representation of others’ states is based on an awareness of how their states affect us - on the basis of how they affected us originally.”* The present finding point to a functional linkage between other- and self-mentalizing suggesting that engagement with others’ mental states may support access to one’s own internal states. This interpretation is consistent with developmental accounts proposing that self-understanding emerges through interactions with others and that the two domains mutually reinforce each other across development (35). Future longitudinal research will be needed to clarify how these reciprocal processes unfold over time.

Interestingly, other-mentalizing was directly and positively related to psychopathology. This finding can be best interpreted in light of evidence linking hypermentalization, defined as the tendency to overattribute mental states to others, to psychopathology, notably found to be a key feature in borderline personality disorder (64–66). It is noteworthy to underline in this context that self-mentalizing showed a strong protective direct effect, through a negative association with psychopathological expression. This result follows other studies showing that the capacity to recognize and regulate one’s own mental states is a crucial buffer against psychological distress, and that self-mentalizing plays a key factor in relation to psychopathology (67–69).

With regards to indirect effects, threat but not deprivation yielded the three significant indirect paths observed in this sample. The first two indirect effects from threat individually go through other-mentalizing, and self-mentalizing, to finally link to psychopathology. These findings are consistent with the neurocognitive social-transactional model proposed by McCrory et al. (70). In line with this model, in this second path, from threat to self-mentalizing to psychopathology, of latent vulnerability, alterations in threat processing following maltreatment indirectly heighten vulnerability to psychopathology through their effects on social cognition and self-representation. Heightened threat sensitivity biases the interpretation of social cues, promoting hostile attribution and maladaptive responses that increase interpersonal stress and reduce the size and quality of social networks. Concurrently, according to authors, threat-related disruptions in autobiographical memory and self-concept undermine reflective capacities and emotion regulation, further limiting adaptive social functioning. These processes together constrain the development of a supportive social architecture, offering a plausible mechanism through which early threat exposure exerts indirect effects on later psychopathology. The final indirect effect, from threat to self-mentalizing to other-mentalizing to psychopathology, translated a serial mediation whereby higher threat predicted greater other-mentalizing, which in turn related to enhanced self-mentalizing and ultimately to lower psychopathology. This path suggests that some individuals may harness social-cognitive vigilance adaptively, using heightened attention to others as a pathway to strengthen self-reflection. This echoes developmental theories proposing that the capacity for self-mentalizing emerges from being mentalized by others (35), as well as the social biofeedback theory of affect mirroring, stating that caregivers help infants construct internal representations of their emotions through marked and contingent reflections of affect (57). When such mirroring is inconsistent, absent, or threatening, the child may internalize distorted self-representations, or the so-called “alien self” (35). An example of defensive adaptations of such trauma responses to illustrate interpersonal trauma following childhood abuse is the identification with the aggressor (71). The pattern found in our results, showing both risk and adaptive elements in the threat-mentalizing link, captures this duality: excessive vigilance toward others can initially serve as a protective mechanism but, when integrated in reflective contexts, may also scaffold a more coherent sense of self. This duality resonates with recent accounts of heterogeneity in trauma responses and mentalizing processes, suggesting that the integration of traumatic experiences can lead either to psychopathology or to adaptive meaning-making, depending on the quality of reflective processing (72). Overall, these findings highlight the complex interplay between early adverse experiences and mentalizing processes, illustrating that threat exposure may not only confer risk but may also engage regulatory processes in which mentalizing others scaffolds understanding of the self, highlighting the interdependence of the two capacities.

Additional analyses revealed that no gender differences emerged in Model 1, indicating similar effects for both men and women. Additionally, age moderation effect was significant in Model 2 as older participants showed higher self-mentalizing and lower psychological distress, and the association between self-mentalizing and psychopathology was weaker at older ages. This supports longitudinal findings that find mentalizing as a significant contributor to narrative self-organization as it develops throughout adolescence and predicts life narrative coherence (73). Critically, we tested an inverse pathway (Model 4) in which threat was linked to psychopathology via self- then other-mentalizing. This alternative pathway did not yield a significant serial indirect effect. Model 1 - from threat to other-mentalizing to self-mentalizing to psychopathology - remained the only supported sequential association. Thus, the additional analyses indicate that the relationship between the two mentalizing domains is not interchangeable: attention to others appears to precede and support access to one’s own internal states rather than the reverse in the context of threatening experiences. This pattern may reflect an adaptive regulatory process whereby social-cognitive vigilance contributes to self-reflective functioning; however, given the cross-sectional nature of the data, conclusions about developmental ordering or causal direction remain tentative.

Clinically, these findings reinforce the rationale for interventions that target mentalizing within safe relational contexts, considering the distinction and inter-connection of self- and other-mentalizing processes. These findings suggest that increasing other-focused mentalization may not, in itself, be an adaptive target for intervention among individuals exposed to threat. Instead, the emphasis should be placed on fostering balanced and context-appropriate social-cognitive functioning. The positive association observed between other-mentalizing and psychopathology may reflect hypervigilant or dysregulated forms of social cognition. From this perspective, interventions should move beyond simply enhancing social understanding and instead aim to cultivate flexible, regulated, and context-sensitive mentalizing capacities. Notably, self-mentalizing process, although sustained by mentalizing others, is crucial to address for its strong protective link to psychopathology, confirming its role as a crucial factor as found related to personality disorders (74) or in transdiagnostic samples (44). Furthermore, in relation to other-mentalizing, findings further support the conceptualization of hypermentalizing to general psychopathological expression (65).

Several limitations should be acknowledged. First, using the CTQ to operationalize deprivation represents a conceptual simplification. Although the neglect subscales capture emotional and physical unavailability, they do not cover the full range of environmental deprivation defined in dimensional models of adversity which also include reduced linguistic, cognitive, and social input (3). The CTQ thus serves as a proxy rather than a comprehensive index of deprivation. Future research should incorporate objective indicators such as parental education, home complexity, or language exposure to best capture this construct and potentially reveal stronger links with self-mentalizing capacities. Second, other dimensions of adversity, such as unpredictability, were not included and may also influence developmental and mentalizing outcomes (4). Third, several associations showed non-linear patterns, which may further introduce measurement error (75). Fourth, cognitive covariates such as IQ and verbal ability were not controlled for and including them would clarify whether the observed effects are specific to mentalizing processing. Fifth, the cross-sectional design precludes causal inference. In addition, the sample reported overall moderate levels of psychological distress as assessed by the Global Severity Index. As a result, the present findings may not generalize to clinical populations characterized by more severe or specific forms of psychopathology, such as individuals diagnosed with personality disorders. Moreover, the use of a global index of distress, while appropriate for capturing overall symptom burden in a community sample, represents a relatively broad and non-specific measure of psychopathology and should be considered a limitation of the study. Additionally, a further limitation of the present study concerns the use of retrospective self-report measures of childhood adversity. Instruments such as the CTQ are inherently subject to recall bias, particularly in samples spanning a wide age range, like the one found in the present study, as the accuracy of retrospective reporting may vary across adulthood. Age-related differences in recall accuracy cannot be fully ruled out in the present design. Future studies relying on prospective or longitudinal approaches will be essential to more precisely capture developmental trajectories of childhood adversity and their associations with mentalization and psychological distress.

In summary, this study provides nuanced evidence that threat and deprivation differentially shape mentalizing and psychopathology. Deprivation limits mentalizing of others, whereas threat disrupts both self- and other-mentalizing, combining hypervigilance with self-disconnection. Across pathways, self-mentalizing emerged as the strongest protective correlate of psychological health, particularly in younger adults. These findings suggest that understanding oneself develops through, and remains interconnected with, understanding others. Integrating dimensional models of adversity with the mentalization framework offers a developmental account of vulnerability and resilience, guiding therapeutic approaches aimed at restoring mentalizing and relational security in individuals affected by early adversity. These findings highlight the need for future longitudinal research to clarify the temporal and potentially reciprocal relationships between self- and other-oriented mentalizing. While the present cross-sectional design identifies associative patterns between these dimensions, longitudinal approaches will be necessary to determine their developmental sequencing and directional dynamics, particularly in relation to psychological distress. More broadly, these findings illustrate how early adversity may recalibrate social-cognitive systems in ways that shape both vulnerability and resilience across the lifespan.

## Data Availability

The datasets presented in this article are not readily available due to ethical restrictions. Requests to access the datasets should be directed to the corresponding author(s).

## Group members of RF-TBM Consortium

Jallal Benmiloud, Margaux Bouteloup, Pablo Cascone, Paco Prada and Mario Speranza.

## References

[1] Schöbi B Holmer P Rapicault A Schöbi D . Bestrafungsverhalten von Eltern in der Schweiz. In: Eine wissenschaftliche Begleitung der Präventionskampagne «Starke Ideen–Es gibt immer eine Alternative zur Gewalt». Fribourg: oO: Institut für Familienforschung und-beratung der Universität Freiburg (2020).

[2] Felitti VJ Anda RF Nordenberg D Williamson DF Spitz AM Edwards V . Relationship of childhood abuse and household dysfunction to many of the leading causes of death in adults. The adverse childhood experiences (ACE) study. Am J Prev Med. (1998) 14:245–58. doi: 10.1016/s0749-3797(98)00017-8 9635069

[3] McLaughlin KA Sheridan MA Lambert HK . Childhood adversity and neural development: Deprivation and threat as distinct dimensions of early experience. Neurosci Biobehav Rev. (2014) 47:578–91. doi: 10.1016/j.neubiorev.2014.10.012 25454359 PMC4308474

[4] Sheridan MA McLaughlin KA . Dimensions of early experience and neural development: deprivation and threat. Trends Cognit Sci. (2014) 18:580–5. doi: 10.1016/j.tics.2014.09.001 25305194 PMC4252647

[5] Lyons-Ruth K Jacobvitz D . Attachment disorganization: Genetic factors, parenting contexts, and developmental transformation from infancy to adulthood. In: Handbook of attachment: theory, research, and clinical applications, 2nd ed. New York: The Guilford Press (2008). p. 666–97.

[6] McLaughlin KA Weissman D Bitrán D . Childhood adversity and neural development: A systematic review. Annu Rev Dev Psychol. (2019) 1:277–312. doi: 10.1146/annurev-devpsych-121318-084950 32455344 PMC7243625

[7] Teicher MH Samson JA . Annual research review: Enduring neurobiological effects of childhood abuse and neglect. J Child Psychol Psychiatry. (2016) 57:241–66. doi: 10.1111/jcpp.12507 26831814 PMC4760853

[8] Stefanovic M Ehring T Wittekind CE Kleim B Rohde J Krüger-Gottschalk A . Comparing PTSD symptom networks in type I vs. type II trauma survivors. Eur J Psychotraumatol. (2022) 13:2114260. doi: 10.1080/20008066.2022.2114260 36186163 PMC9518442

[9] Terr LC . Childhood traumas: an outline and overview. Am J Psychiatry. (1991) 148:10–20. doi: 10.1176/ajp.148.1.10 1824611

[10] Nestgaard Rød Å Schmidt C . Complex PTSD: what is the clinical utility of the diagnosis? Eur J Psychotraumatol. (2021) 12:2002028. doi: 10.1080/20008198.2021.2002028 34912502 PMC8667899

[11] Shevlin M Hyland P Roberts NP Bisson JI Brewin CR Cloitre M . A psychometric assessment of disturbances in self-organization symptom indicators for ICD-11 complex PTSD using the International Trauma Questionnaire. Eur J Psychotraumatol. (2018) 9:1419749. doi: 10.1080/20008198.2017.1419749 29372014 PMC5774393

[12] Herman JL . Complex PTSD: A syndrome in survivors of prolonged and repeated trauma. J Traumatic Stress. (1992) 5:377–91. doi: 10.1007/BF00977235 30311153

[13] Kliethermes M Schacht M Drewry K . Complex trauma. Child Adolesc Psychiatr Clinics. (2014) 23:339–61. doi: 10.1016/j.chc.2013.12.009 24656584

[14] Martalek A Dubertret C Fovet T Le Strat Y Tebeka S . Distressing memories: A continuum from wellness to PTSD. J Affect Disord. (2024) 363:198–205. doi: 10.1016/j.jad.2024.07.076 39029679

[15] van der Kolk B . Developmental trauma disorder: Toward a rational diagnosis for children with complex trauma histories. Psychiatr Ann. (2005) 35:401–8. doi: 10.3928/00485713-20050501-06

[16] Hovens JGFM Giltay EJ Wiersma JE Spinhoven P Penninx BWJH Zitman FG . Impact of childhood life events and trauma on the course of depressive and anxiety disorders. Acta Psychiatrica Scandinavica. (2012) 126:198–207. doi: 10.1111/j.1600-0447.2011.01828.x 22268708

[17] Wang SK Feng M Fang Y Lv L Sun GL Yang SL . Psychological trauma, posttraumatic stress disorder and trauma-related depression: A mini-review. World J Psychiatry. (2023) 13:331–9. doi: 10.5498/wjp.v13.i6.331 37383283 PMC10294137

[18] Wei X Lü W . Childhood trauma and internalizing and externalizing behavior problems among adolescents: Role of executive function and life events stress. J Adolescence. (2023) 95:740–50. doi: 10.1002/jad.12150 36751143

[19] Begemann MJH Schutte MJL van Dellen E Abramovic L Boks MP van Haren NEM . Childhood trauma is associated with reduced frontal gray matter volume: A large transdiagnostic structural MRI study. Psychol Med. (2023) 53:741–9. doi: 10.1017/S0033291721002087 34078485 PMC9975993

[20] Hogg B Gardoki-Souto I Valiente-Gómez A Rosa AR Fortea L Radua J . Psychological trauma as a transdiagnostic risk factor for mental disorder: An umbrella meta-analysis. Eur Arch Psychiatry Clin Neurosci. (2023) 273:397–410. doi: 10.1007/s00406-022-01495-5 36208317

[21] McLaughlin KA Colich NL Rodman AM Weissman DG . Mechanisms linking childhood trauma exposure and psychopathology: A transdiagnostic model of risk and resilience. BMC Med. (2020) 18:96. doi: 10.1186/s12916-020-01561-6 32238167 PMC7110745

[22] Bateman A Fonagy P . Handbook of mentalizing in mental health practice Arlington, VA: American Psychiatric Publishing, Inc. (2012).

[23] Fonagy P Luyten P . A developmental, mentalization-based approach to the understanding and treatment of borderline personality disorder. Dev Psychopathol. (2009) 21:1355–81. doi: 10.1017/s0954579409990198 19825272

[24] Luyten P Campbell C Allison E Fonagy P . The mentalizing approach to psychopathology: State of the art and future directions. Annu Rev Clin Psychol. (2020) 16:297–325. doi: 10.1146/annurev-clinpsy-071919-015355 32023093

[25] Yang L Huang M . Childhood maltreatment and mentalizing capacity: A meta-analysis. Child Abuse Negl. (2024) 149:106623. doi: 10.1016/j.chiabu.2023.106623 38245975

[26] Bateman A Rüfenacht E Perroud N Debbané M Nolte T Shaverin L . Childhood maltreatment, dissociation and borderline personality disorder: Preliminary data on the mediational role of mentalizing in complex post-traumatic stress disorder. Psychol Psychother. (2024) 97 Suppl 1:58–74. doi: 10.1111/papt.12514 38108566

[27] Rüfenacht E Shaverin L Stubley J Smits ML Bateman A Fonagy P . Addressing dissociation symptoms with trauma-focused mentalization-based treatment. Psychoanalytic Psychother. (2023) 37:467–91. doi: 10.1080/02668734.2023.2272765 37339054

[28] Chelouche-Dwek G Fonagy P . Mentalization-based interventions in schools for enhancing socio-emotional competencies and positive behaviour: A systematic review. Eur Child Adolesc Psychiatry. (2025) 34:1295–315. doi: 10.1007/s00787-024-02578-5 39264381 PMC12000265

[29] Bateman A Fonagy P Campbell C Luyten P Debbané M . Cambridge guide to mentalization-based treatment (MBT). Cambridge: Cambridge University Press (2023).

[30] Dimitrijević A Hanak N Altaras Dimitrijević A Jolić Marjanović Z . The mentalization scale (MentS): A self-report measure for the assessment of mentalizing capacity. J Pers Assess. (2018) 100:268–80. doi: 10.1080/00223891.2017.1310730 28436689

[31] Fonagy P Allison E . The role of mentalizing and epistemic trust in the therapeutic relationship. US: Educational Publishing Foundation (2014). doi: 10.1037/a0036505 24773092

[32] Campbell C Allison E . Mentalizing the modern world. Psychoanalytic Psychother. (2022) 36:206–17. doi: 10.1080/02668734.2022.2089906 37339054

[33] Schwarzer N-H Behringer N Dees P Gingelmaier S Henter M Kirsch H . Epistemic mistrust mediates the association between childhood maltreatment and impairments in mentalizing in a sample of university students. Child Abuse Negl. (2025) 163:107436. doi: 10.1016/j.chiabu.2025.107436 40168917

[34] Ensink K Normandin L Target M Fonagy P Sabourin S Berthelot N . Mentalization in children and mothers in the context of trauma: An initial study of the validity of the child reflective functioning scale. Br J Dev Psychol. (2015) 33:203–17. doi: 10.1111/bjdp.12074 25483125

[35] Fonagy P Gergely G Jurist EL Target M . Affect regulation, mentalization, and the development of the self. New York: Other Press (2002).

[36] McLaughlin KA Sheridan MA Winter W Fox NA Zeanah CH Nelson CA . Widespread reductions in cortical thickness following severe early-life deprivation: A neurodevelopmental pathway to attention-deficit/hyperactivity disorder. Biol Psychiatry. (2014) 76:629–38. doi: 10.1016/j.biopsych.2013.08.016 24090797 PMC3969891

[37] Murray RJ Schaer M Debbané M . Degrees of separation: a quantitative neuroimaging meta-analysis investigating self-specificity and shared neural activation between self-and other-reflection. Neurosci Biobehav Rev. (2012) 36:1043–59. doi: 10.1016/j.neubiorev.2011.12.013 22230705

[38] Murray RJ Debbané M Fox PT Bzdok D Eickhoff SB . Functional connectivity mapping of regions associated with self‐and other‐processing. Hum Brain Mapp. (2015) 36:1304–24. doi: 10.1002/hbm.22703 25482016 PMC4791034

[39] Tironi M Charpentier Mora S Liotti M Fiorini Bincoletto A Tanzilli A Cavanna D . Adverse childhood experiences and psychological maladjustment in adolescence: The protective role of epistemic trust, mentalized affectivity, and reflective functioning. J Clin Psychol. (2024) 80:2228–46. doi: 10.1002/jclp.23733 39101491

[40] Meade AW Craig SB . Identifying careless responses in survey data. psychol Methods. (2012) 17:437–55. doi: 10.1037/a0028085 22506584

[41] Bernstein DP Stein JA Newcomb MD Walker E Pogge D Ahluvalia T . Development and validation of a brief screening version of the childhood trauma questionnaire. Child Abuse Negl. (2003) 27:169–90. doi: 10.1016/s0145-2134(02)00541-0 12615092

[42] Paquette D Laporte L Bigras M Zoccolillo M . Validation de la version française du CTQ et prévalence de l’histoire de maltraitance. Santé Mentale au Québec. (2004) 29:201–20. doi: 10.7202/008831ar 15470573

[43] Bernstein DP Fink L Handelsman L Foote J . Childhood trauma questionnaire. In: Assessment of family violence: A handbook for researchers and practitioners San Antonio: The Psychological Corporation. (1998).

[44] Descartes F Besch V Bouteloup M Nicastro R Pham E Rüfenacht E . Validation of the mentalization scale (MentS) in francophone control and clinical samples. PloS One. (2025) 20:e0332724. doi: 10.1371/journal.pone.0332724 41150688 PMC12561985

[45] Derogatis LR Unger R . Symptom checklist-90-revised. Corsini Encyclopedia Psychol. (2010), 1–2. doi: 10.1002/9780470479216.corpsy0970 41531421

[46] Pariente P Lepine JP Boulenger JP Zarifian E Lemperiere T Lellouch J . The Symptom Check-List 90R (SCL-90R) in a French general psychiatric 708 outpatient sample: Is there a factor structure? Psychiatr Psychobiologie. (1989) 4:151–7. doi: 10.1017/s0767399x00001577 41292463

[47] Hair J Hult GTM Ringle C Sarstedt M . A primer on partial least squares structural equation modeling (PLS-SEM) Los Angeles: SAGE Publications, Inc. (2022).

[48] Vinzi VE Trinchera L Amato S . PLS path modeling: from foundations to recent developments and open issues for model assessment and improvement. Handb Partial Least Squares: Concepts Methods Appl. (2009), 47–82. doi: 10.1007/978-3-540-32827-8_3 11826839

[49] Hair JF Black WC Babin BJ Anderson RE . Multivariate data analysis: a global perspective (7th ed.) Upper Saddle River, New Jersey, USA: Pearson Education (2010).

[50] Tenenhaus M Pages J Ambroisine L Guinot C . PLS methodology to study relationships between hedonic judgements and product characteristics. Food Qual Preference. (2005) 16:315–25. doi: 10.1016/j.foodqual.2004.05.013 38826717

[51] Hair JF . A primer on partial least squares structural equation modeling (PLS-SEM). Los Angeles: sage (2014).

[52] Chin WW . Commentary: issues and opinion on structural equation modeling. MIS Quarterly. (1998).

[53] Ab Hamid MR Sami W Mohmad Sidek M . Discriminant validity assessment: Use of fornell & larcker criterion versus HTMT criterion. J Physics: Conf Ser. (2017) 890(1):012163. doi: 10.1088/1742-6596/890/1/012163

[54] Henseler J Ringle CM Sarstedt M . A new criterion for assessing discriminant validity in variance-based structural equation modeling. J Acad Marketing Sci. (2015) 43:115–36. doi: 10.1007/s11747-014-0403-8 30311153

[55] Kock N . Common method bias in PLS-SEM: A full collinearity assessment approach. Int J e-Collaboration (ijec). (2015) 11:1–10. doi: 10.1007/978-3-319-64069-3_11 28118817 PMC5259953

[56] Freeman C . What is mentalizing? An overview. Br J Psychother. (2016) 32:189–201. doi: 10.1111/bjp.12220 40046247

[57] Gergely G Watson JS . The social biofeedback theory of parental affect-mirroring: The development of emotional self-awareness and self-control in infancy. Int J Psychoanal. (1996) 77 :1181–212. doi: 10.4324/9780429471643-7 9119582

[58] Sheridan MA Zeanah CH . Dimensions of adversity exposure and psychopathology: deprivation and threat. J Am Acad Child Adolesc Psychiatry. (2019) 58:S339–40. doi: 10.1016/j.jaac.2019.07.807 38826717

[59] Caspi A Houts RM Belsky DW Harrington H Hogan S Ramrakha S . Childhood forecasting of a small segment of the population with large economic burden. Nat Hum Behav. (2016) 1:5. doi: 10.1038/s41562-016-0005 28706997 PMC5505663

[60] Luyten P Fonagy P . Mentalising in attachment contexts. In: The routledge handbook of attachment: theory. London: Routledge (2014). p. 107–26.

[61] McCrory EJ Viding E . The theory of latent vulnerability: Reconceptualizing the link between childhood maltreatment and psychiatric disorder. Dev Psychopathol. (2015) 27:493–505. doi: 10.1017/s0954579415000115 25997767

[62] Fonagy P Target M . Attachment and reflective function: Their role in self-organization. Dev Psychopathol. (1997) 9:679–700. doi: 10.1017/s0954579497001399 9449001

[63] Fotopoulou A Tsakiris M . Mentalizing homeostasis: The social origins of interoceptive inference. Neuropsychoanalysis. (2017) 19:3–28. doi: 10.1080/15294145.2017.1294031

[64] Bo S Sharp C Fonagy P Kongerslev M . Hypermentalizing, attachment, and epistemic trust in adolescent BPD: Clinical illustrations. Pers Disord. (2017) 8:172–82. doi: 10.1037/per0000161 26691672

[65] McLaren V Gallagher M Hopwood CJ Sharp C . Hypermentalizing and borderline personality disorder: A meta-analytic review. Am J Psychother. (2022) 75:21–31. doi: 10.1176/appi.psychotherapy.20210018 35099264

[66] Sharp C Pane H Ha C Venta A Patel AB Sturek J . Theory of mind and emotion regulation difficulties in adolescents with borderline traits. J Am Acad Child Adolesc Psychiatry. (2011) 50:563–573.e561. doi: 10.1016/j.jaac.2011.01.017 21621140

[67] Ballespí S Nonweiler J Sharp C Vives J Barrantes-Vidal N . Self- but not other-mentalizing moderates the association between BPD symptoms and somatic complaints in community-dwelling adolescents. Psychol Psychotherapy: Theory Res Pract. (2022) 95:905–20. doi: 10.1111/papt.12409 35746823 PMC9795931

[68] Ballespí S Vives J Nonweiler J Perez-Domingo A Barrantes-Vidal N . Self- but not other-dimensions of mentalizing moderate the impairment associated with social anxiety in adolescents from the general population [original research. Front Psychol. (2021) 12:721584. doi: 10.3389/fpsyg.2021.721584 34790146 PMC8591043

[69] Nonweiler J Torrecilla P Kwapil TR Ballespí S Barrantes-Vidal N . I don’t understand how I feel: mediating role of impaired self-mentalizing in the relationship between childhood adversity and psychosis spectrum experiences [Original Research. Front Psychiatry. (2023) 14:1268247. doi: 10.3389/fpsyt.2023.1268247 38098634 PMC10719857

[70] McCrory E Foulkes L Viding E . Social thinning and stress generation after childhood maltreatment: A neurocognitive social transactional model of psychiatric vulnerability. Lancet Psychiatry. (2022) 9:828–37. doi: 10.1016/S2215-0366(22)00202-4 35926524

[71] Lahav Y Cloitre M Hyland P Shevlin M Ben-Ezra M Karatzias T . Complex PTSD and identification with the aggressor among survivors of childhood abuse. Child Abuse Negl. (2025) 160:107196. doi: 10.1016/j.chiabu.2024.107196 39700595

[72] Berthelot N Garon-Bissonnette J . Characterizing the heterogeneity of disruptions in the resolution of trauma among women exposed to childhood maltreatment. Dev Psychopathol. (2024) 37(3):1–14. doi: 10.1017/S0954579424001019 39291363

[73] Köber C Kuhn MM Peters I Habermas T . Mentalizing oneself: Detecting reflective functioning in life narratives. Attachment Hum Dev. (2019) 21:313–31. doi: 10.1080/14616734.2018.1473886 29768982

[74] Jańczak MO Górska D Jurek P Taubner S . Self-other mentalizing and attachment insecurity in the dimensional model of personality disorders: From research to clinical practice. medRxiv. (2025) 32(6):2025.2001.2002.25319931. doi: 10.1101/2025.01.02.25319931 41217414

[75] Moosbrugger H Schermelleh-Engel K Kelava A Klein A . Testing multiple nonlinear effects in structural equation modeling: A comparison of alternative estimation approaches. (2009) 103–36.

