# Supplementary material for: Associations between childhood threat and deprivation experiences, self- and other-mentalizing, and adult psychopathology: evidence from a community sample
Source: Front Psychiatry. 2026 Jul 17;17:1745165. doi: 10.3389/fpsyt.2026.1745165 (PMC13425142; doi:10.3389/fpsyt.2026.1745165)
Supplement: Supplementary file 1 [file Table1.docx]

Supporting information

RF-TBM Consortium

The following researchers contributed to this work as part of the Réseau Francophone des Thérapies Basées sur la mentalisation (RF-TBM) Consortium:

Mario Speranza

Paris-Saclay University, UVSQ, INSERM, Center for Epidemiology and Population Health Team “DevPsy”, Villejuif, France

University Department of Child and Adolescent Psychiatry, Versailles Hospital Center, Le Chesnay-Rocquencourt, France

Paco Prada

Consultation Liaison and Crisis Intervention, University Hospitals of Geneva, Switzerland

Department of Psychiatry, Geneva University Hospital, Geneva, Switzerland; Department of Medicine, University of Geneva, Geneva, Switzerland

Pablo Cascone

Division of Child and Adolescent Psychiatry, Department of Psychiatry, University Hospital of Lausanne and University of Lausanne, Lausanne, Switzerland

Jalal Belmioud

Office Médico-Pédagogique Research Unit, Department of Psychiatry, University of Geneva School of Medicine, Geneva, Switzerland
