# Supplementary material for: Associations between childhood threat and deprivation experiences, self- and other-mentalizing, and adult psychopathology: evidence from a community sample
Source: Front Psychiatry. 2026 Jul 17;17:1745165. doi: 10.3389/fpsyt.2026.1745165 (PMC13425142; doi:10.3389/fpsyt.2026.1745165)

Supplementary material

**Figure 2**

*Results from permutation analyses for women on Model 1*


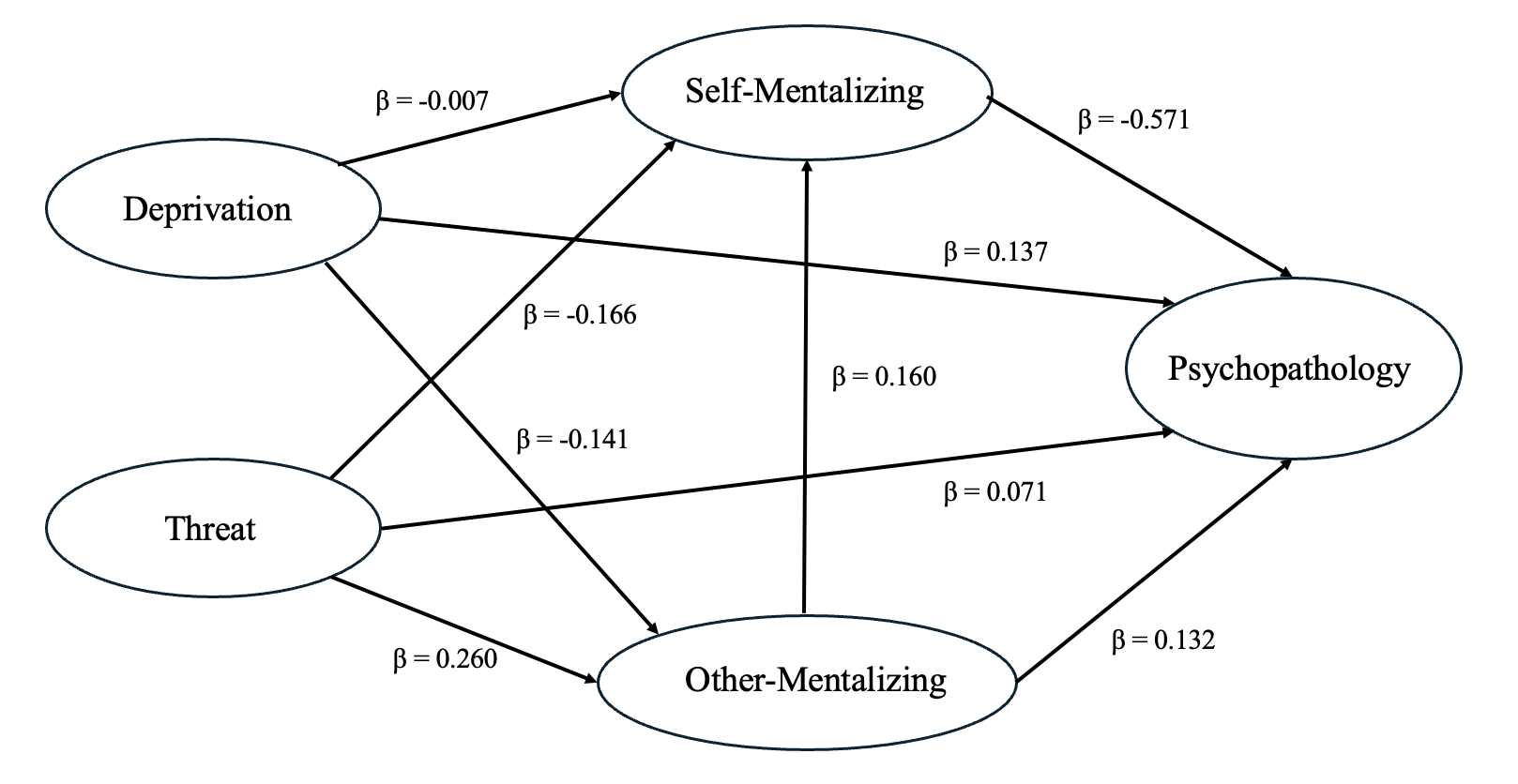


**Figure 3**

*Results from permutation analyses for men on Model 1*


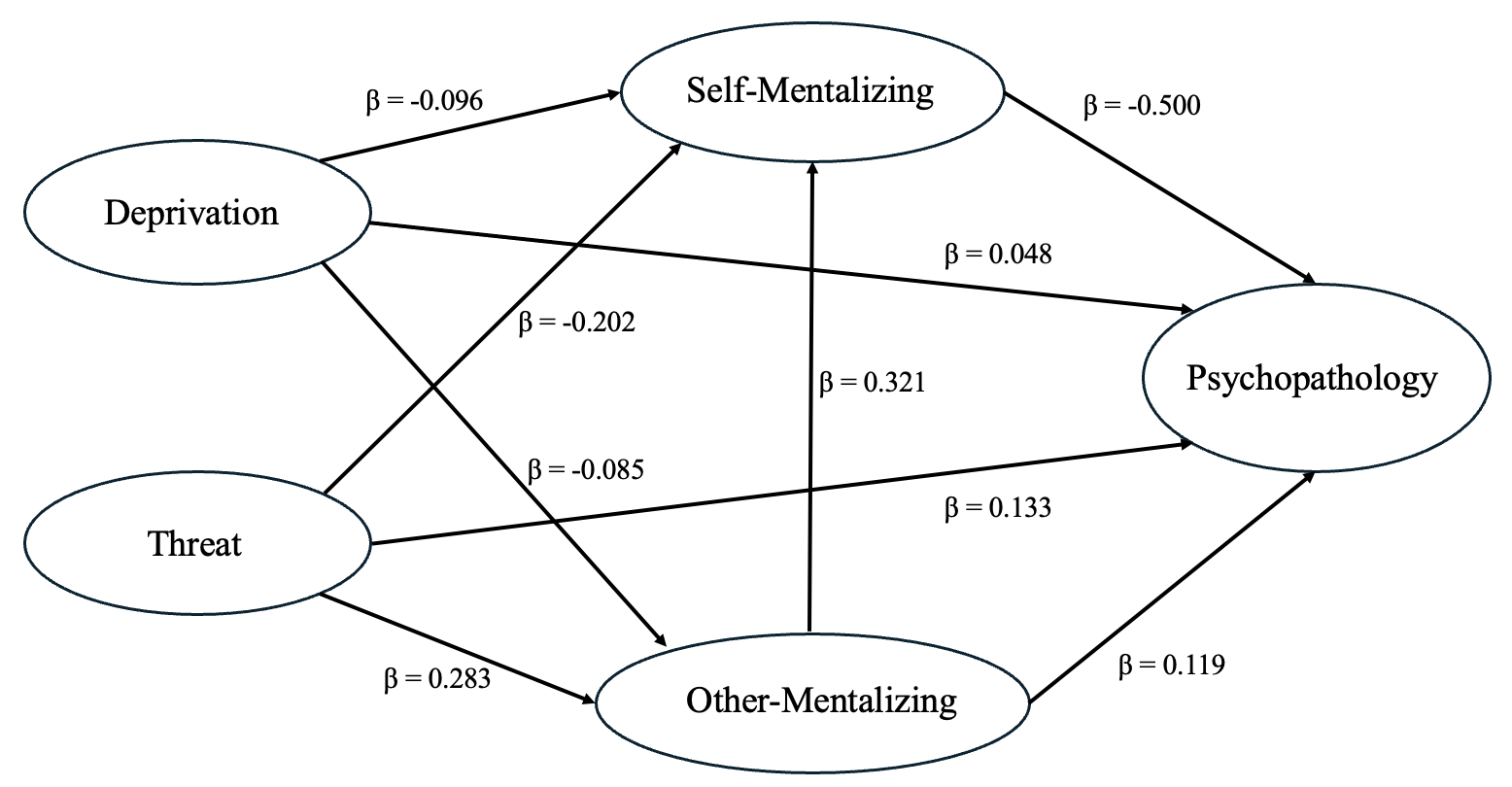


**Figure 4**

*Model 2- Results from the moderating effect of age on the relationship between self-mentalizing and psychopathology*


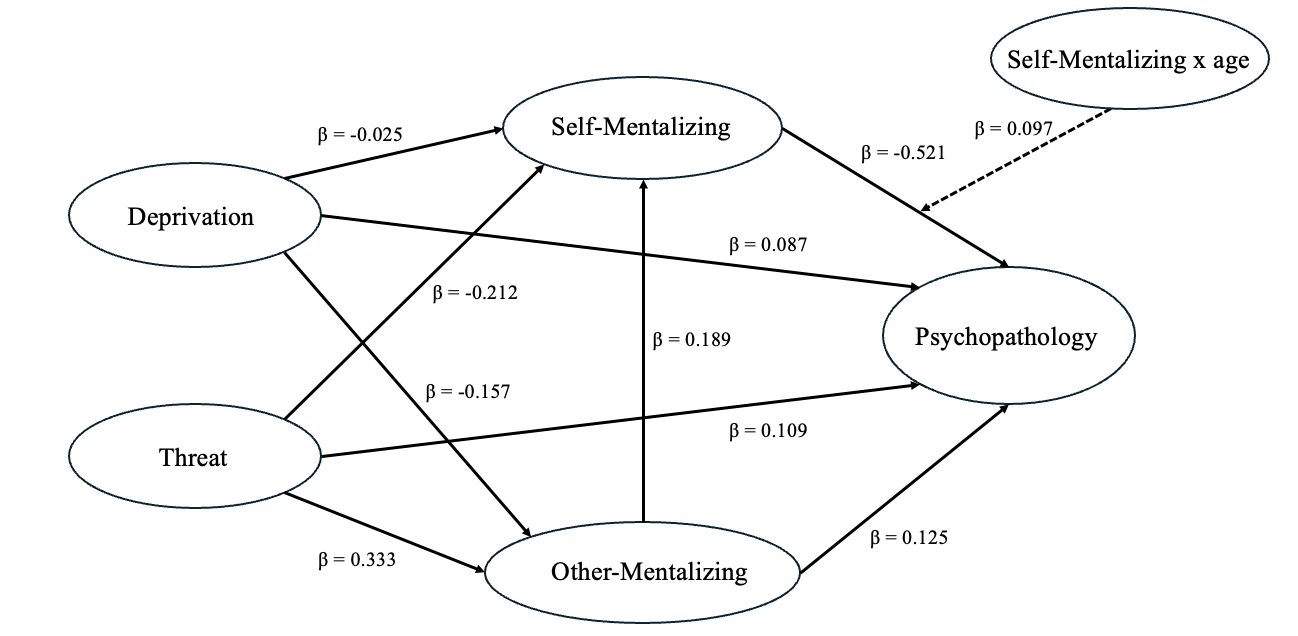


**Figure 5**

*Model 3 - Results from the moderating effect of age on the relationship between other-mentalizing and psychopathology*


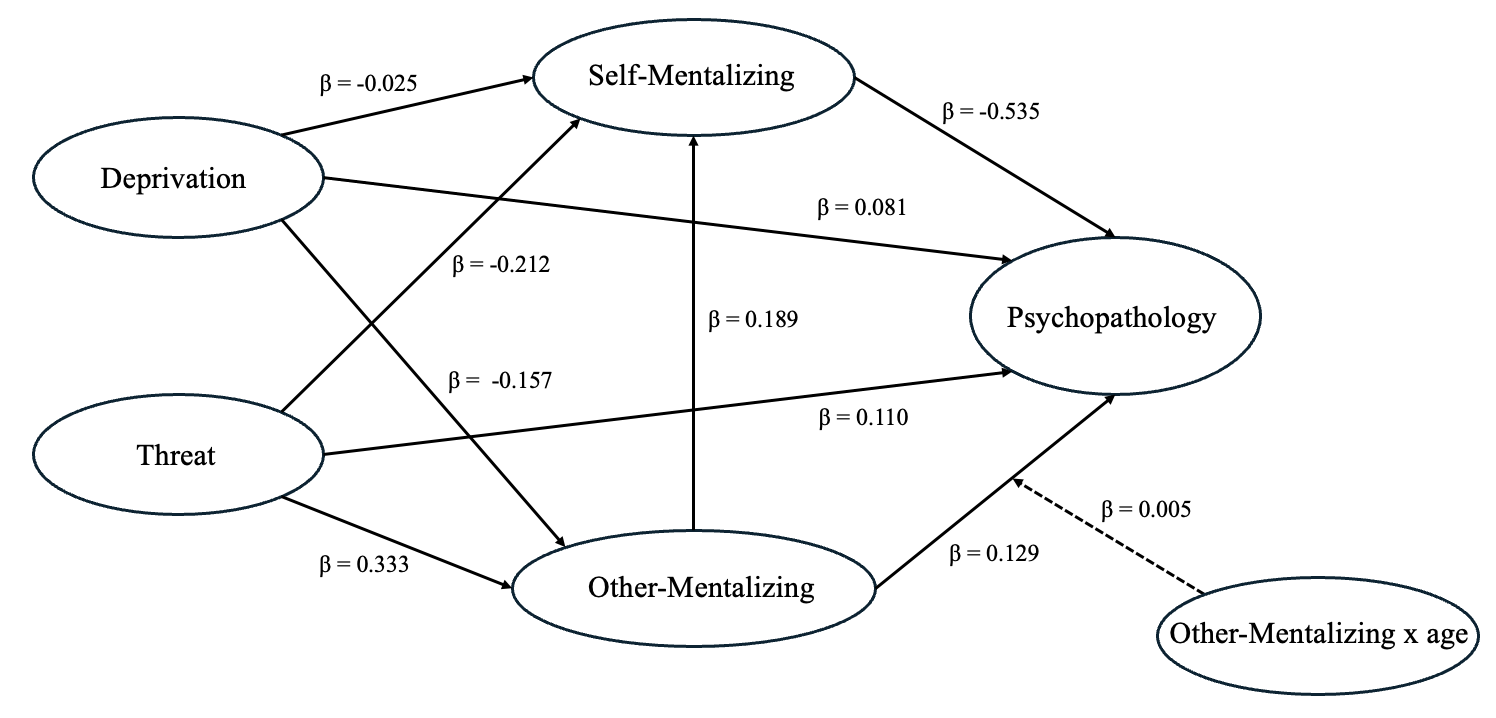


**Figure 5**

*Model 4 - Results from the inverse model*
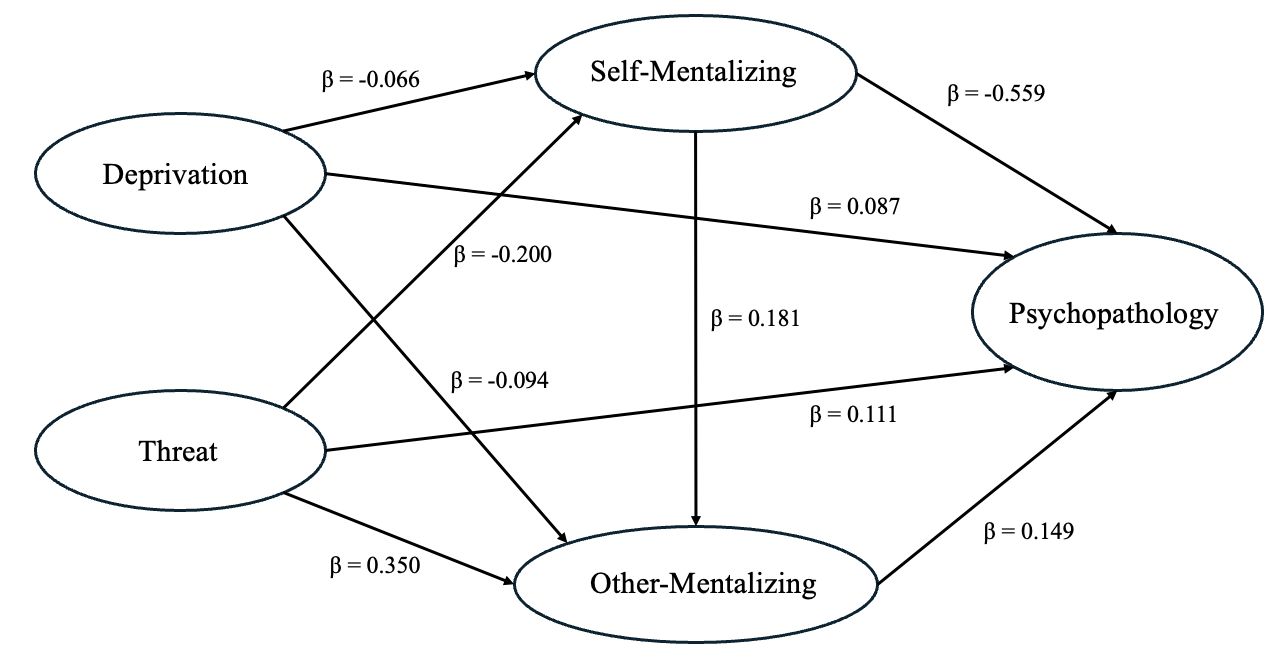

Supplement: Supplementary file 2 [file Table2.docx]
